# Immunogenicity of Sulfated Lactosyl Archaeol Archaeosome-Adjuvanted Versus Non-Adjuvanted SARS-CoV-2 Spike Booster Vaccines in Young and Aged Balb/c Mice

**DOI:** 10.3390/vaccines13121257

**Published:** 2025-12-18

**Authors:** Felicity C. Stark, Bassel Akache, Tyler M. Renner, Gerard Agbayani, Lise Deschatelets, Renu Dudani, Blair A. Harrison, Usha D. Hemraz, Sophie Régnier, Matthew Stuible, Yves Durocher, Michael J. McCluskie

**Affiliations:** 1 Human Health Therapeutics, National Research Council of Canada, Ottawa, ON K1A 0R6, Canada; felicity.stark@nrc-cnrc.gc.ca (F.C.S.); bassel.akache@nrc-cnrc.gc.ca (B.A.);; 2 Human Health Therapeutics, National Research Council of Canada, Montreal, QC H4P 2R2, Canada; 3 Aquatic and Crop Resource Development, National Research Council of Canada, Montreal, QC H4P 2R2, Canada

**Keywords:** archaeosomes, sulfated lactosylarchaeol, SLA, COVID-19, SARS-CoV-2 booster vaccines, infectious diseases, protein subunit vaccines, vaccines in young and aged mice

## Abstract

**Background/Objectives:** The rise of immune escape variants of the SARS-CoV-2 virus has prompted the development of vaccines based on the variant’s spike antigen sequence. Since variant-specific SARS-CoV-2 vaccines are mostly administered as boosters to individuals previously vaccinated with reference (Ref.) strain-based vaccines, a better understanding of their immunogenicity in this context is essential. Protein subunit vaccines have a well-established track record of safety. Herein, we assessed the ability of variant-specific protein subunit vaccine formulations to boost pre-existing Ref. strain-specific immune responses compared to boosting with a Ref. strain-specific formulation in young and aged female Balb/c mice. **Methods:** Following a priming vaccination series with Ref. spike protein adjuvanted with sulfated lactosyl archaeol (SLA) archaeosomes on days 0 and 21, immune responses were evaluated in young and aged female Balb/c mice. On day 91, mice received a third immunization with Ref., Beta, or Delta spike protein formulations, with or without SLA archaeosomes. Antibody titers, neutralization activity, and cellular immune responses were measured to assess the impact of the booster formulation. **Results:** Aged mice exhibited lower antibody titers throughout the study and a decline over time compared to young mice. After a third immunization, responses were boosted by all vaccine formulations (Ref., Beta, or Delta), with or without adjuvant. However, variant-specific antigen formulations did not overcome immune imprinting from the priming series or increase neutralization activity against the corresponding SARS-CoV-2 variants in either age group. **Conclusions:** Variant-specific protein subunit vaccines enhanced immune responses but did not overcome immune imprinting induced by the Ref. strain’s priming. The inclusion of SLA archaeosomes improved cellular immunity, supporting their potential role in optimizing booster vaccine performance, particularly in aged populations.

## 1. Introduction

While mRNA vaccines had a clear advantage during the COVID-19 pandemic with their unparalleled speed of development and production, protein-based vaccine formulations offer several significant advantages that make them highly attractive for pandemic response planning [1]. Unlike mRNA vaccines, protein-based vaccines are typically stable under standard refrigerated temperatures, making them far more accessible in remote communities that lack extreme-cold-storage infrastructure [2,3]. Additionally, protein-based vaccines have a well-established track record of safety and efficacy, supported by a robust manufacturing capacity [4]. Since the approval of the recombinant hepatitis B vaccine, Recombivax HB, in 1986 [5,6], numerous protein-based vaccines have been approved and proven safe for young adults and pediatric and elderly populations [7]. During the pandemic, Nuvaxovid (NVX-CoV2373), Novavax’s adjuvanted protein-based vaccine, showed high levels of efficacy in protecting against COVID-19 [8]. In addition, Vidprevtyn Beta®, developed by Sanofi, is another protein subunit vaccine that has also been approved for clinical use [9].

Despite the development and deployment of several effective vaccines against COVID-19, the emergence of new and more transmissible variants of SARS-CoV-2 remains a threat [10]. The evolution of the SARS-CoV-2 spike protein has been largely driven by immune evasion, significantly reducing the effectiveness of humoral responses primed by first-generation vaccines [11]. As a result, many SARS-CoV-2 vaccine developers have shifted focus towards developing variant vaccines that target these mutations, with a goal to provide protection against new variant SARS-CoV-2 strains. Following the recommendation of the Committee for Medicinal Products for Human Use at the European Medicines Agency, the first clinically available updated vaccines were bivalent, comprising 50% ancestral strain and 50% Omicron (either BA.1 or BA.4/BA.5 lineages), and they have now shifted to monovalent vaccines targeting Omicron JN.1 lineage variant KP.2 [12,13]. Clinical trials demonstrated the enhanced neutralization of the BA.1 variant in individuals previously vaccinated with the Omicron BA.1-adapted BNT162b2 monovalent or bivalent vaccines, while still maintaining effective responses against the ancestral strain [14]. Despite these advancements, reports indicate that these bivalent vaccines often result in outcomes comparable to or only slightly better than vaccines based solely on the ancestral viral spike protein [14,15,16,17,18,19].

This limited improvement may be attributed to “immunological imprinting”, sometimes referred to as the “original antigenic sin”, where the immune system preferentially recalls memory B cell responses generated against previously encountered epitopes rather than mounting new responses to novel variant-specific epitopes [20,21]. Immunological imprinting arises primarily from the preferential activation of primed B cell clones, because the activation threshold for memory B cells is substantially lower than for naïve B cells. Memory B cells possess higher-affinity B cell receptors for conserved epitopes and have a lower activation threshold compared to naïve B cells. When exposed to a variant strain, there is a rapid expansion of these memory B cell clones. While this recall response can provide broad cross-reactivity, it can also suppress the generation of de novo B cell responses to new epitopes, essentially outcompeting them for resources and additionally through epitope masking. Epitope masking occurs when antibodies to conserved epitopes physically block access of variant epitopes to B cell receptors, limiting the recruitment and maturation of B cells specific for variant epitopes. This poses a significant challenge for variant-specific vaccine design [22,23,24]. Interestingly, it has been shown that increased T cell levels can overcome the suppressive effect that pre-existing antibodies have on de novo B cell activation, and while these B cells often exhibit lower antigen specificity [25], they can also lead to a beneficial increased variant-specific neutralizing responses. This is especially important in aged populations as age-related immune changes include a reduction in naïve T cells, reduced T cell help, and the accumulation of age-associated B cells (ABCs), which produce lower-affinity, non-neutralizing antibodies and contribute to weaker vaccine responses [26]. It is believed that exposure to antigenically distinct variants in an immunostimulatory environment that activates T cells has a higher likelihood of stimulating de novo B cell responses, and one group has reported that the most effective BA.1-neutralizing responses originated from de novo B cell responses, circumventing immunological imprinting altogether [27]. However, another study with a SARS-CoV-2-based vaccine has shown that a single booster dose may not be sufficient to overcome immunological imprinting and that a dual-vaccination approach with a variant-based vaccine was required to induce higher neutralization activity towards the corresponding variant [27]. Further studies are required to confirm these observations under various vaccination settings, especially with regard to whether adjuvants in protein subunit vaccines are necessary to boost the immune response and if they can help promote de novo variant-specific B cell responses [28].

In a pandemic response setting, adjuvants serve an important role by not only enhancing immune responses but also allowing for antigen dose sparing, which increases the number of available doses [29,30,31]. Prior to the COVID-19 pandemic, limited manufacturing and regulatory hurdles made it difficult for new adjuvants to reach the market; however, the pandemic showcased the need for new adjuvants, and increased biomanufacturing capacity has fueled the development of numerous new adjuvanted vaccines. Sulfated lactosyl archaeol (SLA) archaeosomes are a vaccine adjuvant shown in various preclinical studies to induce both strong cellular and humoral immune responses to numerous disease antigens in vivo, including hemagglutinin (HA) [32], hepatitis B surface antigen (HBsAg), Hepatitis C virus E1 and E2, rabbit hemorrhagic disease virus (RHDV) VP60, Schistosoma mansoni Cathepsin B (SmCB), and, most recently, the SARS-CoV-2 spike protein [33,34,35]. SLA archaeosomes consist of liposomes generated with the SLA glycolipid (6′-sulfate-β-d-Galp-(1,4)-β-d-Glcp-(1,1)-archaeol) consisting of a sulfated lactose polar head group fused to archaeol. Compared to conventional liposomes, SLA archaeosomes exhibit high thermal and pH stability and low proton permeability, and they do not generate anti-lipid immune responses. These characteristics enable the use of SLA archaeosomes in a repeat boost vaccination setting. In a previous head-to-head comparison study with commercial adjuvants (including TLR3/4/9 agonists, oil-in-water and water-in-oil emulsions, and aluminum hydroxide), liposomes composed of SLA demonstrated strong adjuvant activity that in many cases was superior to other adjuvants tested [36]. It was particularly good at simultaneously inducing both humoral and cellular immune responses, suggesting that it may be a good candidate for generating de novo B cell responses. We have previously demonstrated the immunogenicity and efficacy of an SLA archaeosome-adjuvanted resistin-trimerized spike protein vaccine [33,34]. In a mono- or multivalent homologous prime boost vaccine study with Ref., Beta, or Delta spike protein, it was shown that adjuvanted protein-subunit vaccines elicited higher neutralizing antibody activity with respect to the corresponding variant than a vaccine based on the original Ref. strain, and a multivalent vaccine was able to cross-neutralize all three variant antigens [34]. In this study, we aim to further explore the immunogenicity of these variant-based protein subunit vaccines when used in a booster setting with or without the adjuvant and whether they can help overcome the immunological imprinting of pre-existing reference-based SARS-CoV-2 immunity in young and aged mice.

## 2. Materials and Methods

### 2.1. Mice

Female Balb/c mice (6–8 weeks old) were obtained from Charles River Laboratories (Saint-Constant, QC, Canada). These mice were either allowed to age in-house until they were 19 months old (Aged mice) or used within 4 weeks (Young mice). All mice were housed in the National Research Council of Canada (NRC) small animal facility and cared for in accordance with the Canadian Council on Animal Care guidelines. All animal procedures were approved under protocol 2020.10 by the NRC Human Health Therapeutics Animal Care Committee.

### 2.2. SLA Archaeosome Adjuvant

SLA archaeosomes were prepared from total polar lipids that were extracted from *Halobacterium salinarum* (ATCC 33170). The extracted lipids were hydrolyzed, and the archaeol core was purified as previously described [37]. Using the archaeol core, sulfated lactosylarchaeol (SLA; 6’-sulfate-β-d-Galp-(1,4)-β-d-Glcp-(1,1)-archaeol) was synthesized and formulated into liposomes (archaeosomes), as described in earlier studies [38,39]. SLA archaeosomes were diluted to the desired concentration in phosphate-buffered saline (PBS) and stored at 4 °C until use (1 week).

### 2.3. Spike Protein Antigen

Recombinant, soluble SARS-CoV-2 spike trimer constructs used for immunization, ELISA, and neutralization assays consist of the spike ectodomain fused to human resistin at the C-terminus. Reference, Beta, and Delta spike trimers contain no purification tags and were produced using stably transfected CHO^2353^ stable pools and purified by spike-affinity chromatography exactly as described [40]. Omicron (BA.1) spike trimers include 6-His and dual-Strep tags at the C-terminus (following the resistin sequence) and were produced by transient transfection of CHO^55E1^ cells and purified by sequential IMAC and Strep-affinity chromatography steps, exactly as described [35]. Trimer formation and protein purity were confirmed by UPLC-SEC and SDS-PAGE, as reported in previous publications [35,40]

### 2.4. Immunization and Sample Collection

**Figure 1:**
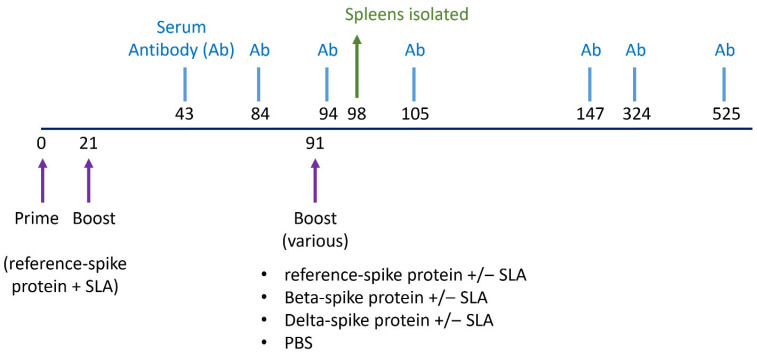
Vaccination and sample collection schedule. Female Balb/c mice aged 8–10 weeks or 19 months were immunized with Ref. spike protein and SLA archaeosomes on day 0 and 21 (*n* = 70) or PBS (*n* = 35). On day 91, mice were randomized into smaller groups (*n* = 5–10) and given different booster vaccinations, including Ref., Beta, or Delta spike protein (with or without SLA archaeosome) or PBS control. Spleens were taken on day 98 to enumerate IFN-γ-producing T cells by ELISPOT (*n* = 5), and blood samples were taken on day 43, 84, 94, 105, 147, 324, and 525 (various sample sizes depending on timepoint; see Table 1).

Spike protein (Ref., Beta, or Delta) and SLA archaeosome adjuvant were diluted in DPBS (Cytiva, Marlborough, MA, USA) prior to administration. Under isoflurane anesthesia, mice were immunized by intramuscular injection (50 µL into the left tibialis anterior muscle) on days 0, 21, and 91 with different vaccine combinations (see Figure 1 and Table 1). Blood was collected from isoflurane anesthetized mice via the submandibular vein on days 43, 84, 94, 105, 147, 324, and 525. A subset of mice was humanely euthanized on day 98 to collect spleens for ELISpot.

### 2.5. Spike-Specific IgG ELISA

Spike-specific total IgG levels in serum were measured by ELISA as described previously [41]. Briefly, 96-well high-binding ELISA plates (Thermo Fisher Scientific, Mississauga, ON, Canada) were coated overnight with spike protein (0.3 µg/mL in PBS), washed, and blocked with 10% fetal bovine serum (FBS; Thermo Fisher Scientific, Mississauga, ON, Canada) for 1 h at 37 °C. Serially diluted serum samples were added, incubated for 1 h, and detected using goat anti-mouse IgG-HRP (1:4000, Southern Biotech, Birmingham, AL, USA). After adding OPD substrates (Sigma-Aldrich/MilliporeSigma, St. Louis, MO, USA) and stopping the reaction with 4N H_2_SO_4_, absorbance was read at 450 nm. IgG titers were defined as the dilution producing an OD 450 of 0.2, calculated with XLfit software, version 5 (ID Business Solutions, Guildford, UK). Samples below this threshold were assigned the lowest tested dilution (i.e., 10) for analysis purposes.

### 2.6. Vero E6 Cell-Based Surrogate SARS-CoV-2 Neutralization Assay

This surrogate neutralization assay was performed similarly to previously described [42]. Briefly, a surrogate neutralization assay was used to assess the ability of serum to block SARS-CoV-2 spike protein binding to Vero E6 cells. Biotinylated spike proteins (Ref., Beta, Delta, or Omicron) were incubated with diluted serum and cells. The geometric mean fluorescent intensity (GMFI) of PE for single cells in each well is measured using flow cytometry. Neutralization was calculated based on a reduction in spike binding (measured as fluorescence intensity) compared to positive and negative controls, according to the formula below. The background control sample includes the GMFI of PE for an average of replicates for cells incubated only with Streptavidin-PE and without spike protein. Positive control samples include the GMFI of PE for an average of replicates for cells incubated with spike protein without serum.

(1) $$\%\;Neutralization=100-\left[100\times \frac{GMFI\;Sample-GMFI\;Background}{GMFI\;Maximum-GMFI\;Background}\right]$$

### 2.7. ELISpot

Spike-specific T cell responses were measured via ELISpot, as described previously [43], using the mouse IFN-γ kit (Cat:3321-4HPW-2; capture antibody: AN18; detection antibody: R4-6A2; Mabtech, Nacka Strand, Sweden). Splenocytes were isolated from mechanically minced spleens in RPMI medium supplemented with 10% FBS, 1% penicillin/streptomycin, 1% glutamine, and 55 μM 2-Mercaptoethanol (all from Thermo Fisher Scientific, Mississauga, ON, Canada). Cells were filtered through a 70 μm cell strainer and counted (Cellometer, Nexcelom, Lawrence, MA, USA). Splenocytes (4 × 10^5^ per well) were stimulated in duplicate with reference SARS-CoV-2 spike peptide pools (JPT Peptide Technologies GmbH, Berlin, Germany) at 2 μg/mL per peptide for ~20 h at 37 °C, 5% CO_2_. Plates were developed per manufacturer’s instructions using AEC substrate (Becton Dickinson, Franklin Lakes, NJ, USA), and spots were counted (Cellular Technology Ltd., Beachwood, OH, USA). The background values were subtracted to calculate antigen-specific IFN-γ+ spot-forming cells (SFCs)/10^6^ splenocytes per animal.

### 2.8. Statistical Analysis

Data were analyzed using GraphPad Prism^®^ version 9 (GraphPad Software, San Diego, CA, USA). Statistical significance was determined by one-way or two-way analysis of variance (ANOVA) followed by post hoc analysis using Tukey’s (comparison across all groups) multiple-comparison test or by a *t*-test, as indicated in the figure legends. Differences with *p* > 0.05 were considered not significant. Significance levels were represented as * *p* < 0.05, ** *p* < 0.01, *** *p* < 0.001, and **** *p* < 0.0001.

## 3. Results

### 3.1. Kinetics of Spike-Specific IgG Antibody Response in Young and Aged Immunized Mice

To determine the optimal timing for subsequent booster studies, we first assessed the kinetics of spike-specific IgG responses in young (8–10 weeks old) and aged (19 months) mice (*n* = 10 per group). The mice were immunized intramuscularly with 2 µg of Ref. spike protein and 1 mg of SLA archaeosomes on days 0 and 21. Systemic immune responses were measured in the serum on days 43, 84, 94, 105, 147, and 324.

**Figure 2:**
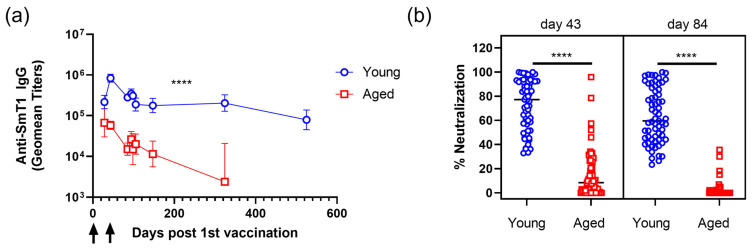
Spike-specific IgG titer kinetics and neutralization after priming immunization series in young and aged mice. Female Balb/c mice aged 8–10 weeks (Young) or 19 months (Aged) were immunized intramuscularly with Ref. spike protein and SLA archaeosomes on days 0 and 21 (indicated with black arrows). Blood samples were collected at various timepoints, and serum was assessed for spike-specific IgG titers (**a**) and % neutralization of Ref. spike protein cellular binding (**b**). See Table 1 for sample sizes, as mice were given different booster vaccinations on day 91; treatment groups prior to day 91 contained 75 mice, and after day 91, they contained 10 mice. Panel (**a**): Data is presented as geometric means, 95% CIs are displayed, and data were analyzed using a mixed-effects model (REML): two-way ANOVA; **** *p* < 0.0001. Panel (**b**): Individual mouse data are displayed, and data were analyzed using a one-way ANOVA with Tukey’s multiple-comparison test; **** *p* < 0.0001.

Spike-specific IgG titers were significantly higher in young mice compared to aged mice by at least 10 fold at all timepoints after the second immunization (Figure 2a). In young mice, spike-specific IgG titers remained relatively stable, with geometric mean titers (GMTs) of 187,668, 177,544, and 203,812 on days 105, 147, and 324, respectively. In contrast, the GMTs in aged mice declined over the same period from 20,013 to 11,469 and then to 2396 (Figure 2a). By day 324 post-first immunization, young mice had a GMT that was 100-fold greater than that observed in aged mice. Young immunized mice were further monitored until 525 days post-first immunization (aged 19.5 months). Although GMTs waned to 139,205, this level was still significantly higher than at any timepoint for mice immunized at 19 months of age.

While the induction of spike-specific IgG antibodies correlates well with vaccine efficacy against COVID-19 [44], the functionality of these antibodies to neutralize their target is an especially important correlate. Using a surrogate cell-based neutralization assay, which has shown a strong correlation to viral-based SARS-CoV-2 assays [33,35], we measured the ability of serum antibodies from all young and aged mice to prevent the binding of spike protein to the ACE2 receptor on the surface of VERO E6 cells (Figure 2b). As we were looking at timepoints prior to the day 91 booster, the sera from all the mice included in the study were analyzed. The serum from young immunized mice exhibited strong neutralizing activity, which waned from 73% on day 43 to 63% on day 84 (*p* < 0.05). In contrast, aged, immunized mice showed significantly lower neutralizing activity than their younger counterparts on both day 43 (*p* < 0.0001) and day 84 (*p* < 0.0001), with a more pronounced decline from 15% on day 43 to 2% on day 84 (Figure 2b).

### 3.2. Spike-Specific IgG Antibody Response in Mice Following Variant Booster Vaccination

**Figure 3:**
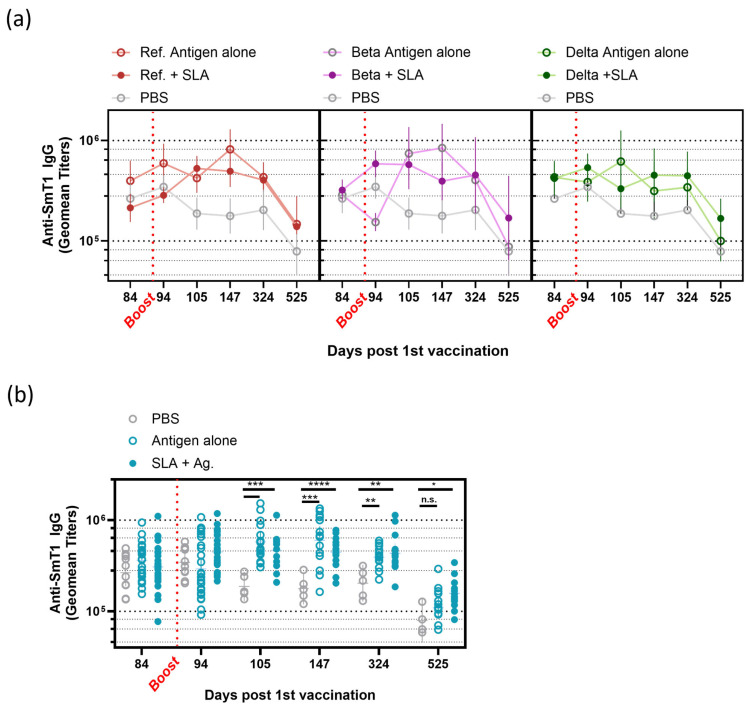
SLA adjuvant does not enhance antibody responses early on but may prolong antibody longevity. Female Balb/c mice aged 8–10 weeks were immunized intramuscularly with Ref. spike protein and SLA archaeosomes on days 0 and 21. Mice were boosted on day 91 with PBS (grey empty circles), Ref., Beta, or Delta spike protein with or without SLA archaeosomes, as indicated on the graph. Blood samples were collected at various timepoints, as indicated in the legend, and serum was assessed for Ref. spike-specific antibodies. (**a**) IgG titers are shown for Ref., Beta, or Delta immunized mice with or without SLA archaeosomes (data are presented as geometric titers ± 95% CI). (**b**) IgG titers are shown for antigen-alone, SLA archaeosomes + antigen, or PBS-immunized mice (individual mouse data are presented; Ref., Beta, and Delta groups are pooled for comparison). A mixed-effects analysis was conducted with Tukey’s multiple-comparison test. If not indicated in the graph, then the comparison was not significant (n.s.). N.s.: *p* > 0.05. * *p* < 0.05, ** *p* < 0.01, *** *p* < 0.005, and **** *p* < 0.001.

**Figure A1:**
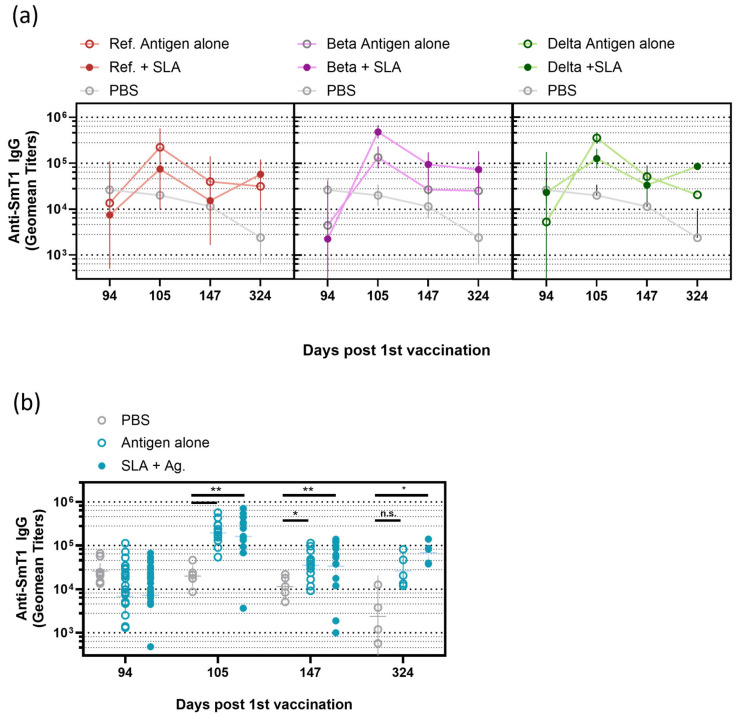
In aged mice, SLA adjuvant does not enhance antibody responses early on but may prolong antibody longevity. Female Balb/c mice aged 19 months were immunized intramuscularly with Ref. spike protein and SLA archaeosomes on days 0 and 21. Mice were boosted on day 91 with PBS (grey empty circles) and Ref., Beta, or Delta spike protein with or without SLA archaeosomes, as indicated on the graph. Blood samples were collected at various timepoints as indicated in the legend, and serum was assessed for Ref.-spike-specific antibodies. (**a**) IgG titers are shown for Ref.-, Beta-, or Delta-immunized mice with or without SLA archaeosomes (data are presented as geometric ± 95% CI). (**b**) Combined IgG titers are shown for antigen-alone, SLA archaeosomes + antigen, or PBS immunized mice. A mixed-effects analysis was conducted with Tukey’s multiple-comparison test. If not indicated in the graph, then the comparison was n.s. N.s.: *p* > 0.05. * *p* < 0.05 and ** *p* < 0.01.

On day 91, cohorts of mice previously immunized with Ref. spike protein and SLA archaeosomes on days 0 and 21 received different variant booster immunizations. These variant boosters included Ref., Beta, or Delta spike antigens, administered alone or combined with SLA archaeosomes (Figure 1). Regardless of vaccine formulation, all mice immunized on day 91 (except those receiving PBS control), both young (Figure 3) and aged (Figure A1a,b), exhibited a significant increase in spike-specific GMTs.

For ELISA assays, Ref. spike was used as the coating antigen for all groups regardless of the identity of the boosting antigen due to previous studies indicating that similar titers were measured whether the coating antigen corresponded to the vaccine antigen or to the Ref. strain [34]. This is not surprising, as ELISA measures antibodies directed against the entire spike protein, and there are a small number of differences in amino acid sequences between the SARS-CoV-2 spike protein of these variants. In contrast, the same publication illustrated that neutralization assays more accurately captured the nuances of variant-specific antibody responses; more specifically, it measures antibodies that are able to inhibit spike binding to ACE2.

Notably, by day 147, young mice boosted with Ref. or Beta spike protein alone achieved the highest overall GMT (upper and lower 95% CI) values of 817,895 (516,185 and 1,295,956) and 840,542 (482,566 and 1,464,071), respectively, significantly surpassing Delta spike protein alone at 315,415 (162,306 and 612,957) (*p* < 0.05). Interestingly, these two groups also surpassed all three SLA-adjuvanted vaccine formulations (Ref: 495,456 (345,503 and 710,492); Beta: 393,531 (254,109 and 609,449); Delta: 452,822 (245,092 and 836,613), but the difference was not statistically significant (Figure 3a). As observed, 19 months after initial vaccination at day 525, the titers among all mice had waned. Anti-spike IgG geometric titers for PBS, Beta antigen alone, or Delta antigen alone waned the most to 78,951 (45,246 and 137,765), 87,718 (45,123 and 170,524), and 100,495 (62,845 and 160,702), respectively. Interestingly, antibody titers in mice that were boosted with Ref antigen alone waned to a lesser extent, 145,505 (78,026 and 278,853), similarly to SLA Ref.-, Beta-, and Delta-boosted groups at 139,205 (116,770 and 165,949), 169,404 (64,659 and 443,883), and 168,163 (106,921 and 264,482), respectively. Although not statistically significant across all timepoints, we observed that a Ref. spike antigen booster, but not Beta or Delta antigen booster, sustained anti-spike IgG titers similar to SLA-adjuvanted groups (Figure 3a).

When antigen-alone groups were pooled and compared to pooled SLA + antigen groups, no significant differences were observed (Figure 3b) at 2-, 8-, or 33-weeks post-booster vaccination (days 105, 147, and 324). By day 525, as the mice approached old age (19 months old), the inclusion of SLA archaeosome adjuvant in the booster immunization led to a slightly more sustained antibody response (Figure 3b). Mice that received the protein antigen-alone booster had a statistically similar GMT to PBS (109,148 and 78,952, respectively), whereas the SLA archaeosome-adjuvanted boosters had a GMT of 157,518, significantly higher than PBS control (*p* < 0.05) (Figure 3b).

### 3.3. Spike-Specific Cross-Neutralization Response in Immunized Mice

**Figure 4:**
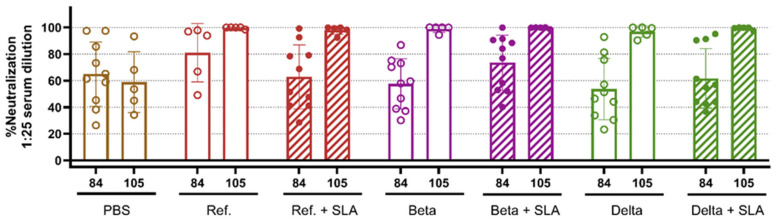
Antigen-alone vaccination elicits potent neutralizing responses against Ref. spike without adjuvant. Female Balb/c mice aged 8–10 weeks were immunized with Ref. spike protein and SLA archaeosomes on days 0 and 21. On day 91, mice were immunized with the vaccine formulation, as indicated on the graph, or PBS control. Blood samples were collected at days 43, 84, and 105 and assessed for % neutralization against spike (Ref.) using a surrogate cell-based neutralization assay; bars are presented as mean with individual data points scattered.

To evaluate the functionality of antibodies induced after variant-booster vaccination and their ability to neutralize spike protein binding to the cellular receptor, we employed a surrogate cell-based neutralization assay. Serum samples collected on days 84 (1 week prior to the booster) and 105 (2 weeks following the booster) were tested for their ability to neutralize Ref. spike protein (Figure 4). By day 105, two weeks after booster immunization, all vaccine formulations induced over 96% neutralization, a significant increase compared to PBS control, which showed 59% neutralization at the same timepoint (*p* < 0.0001, Figure 4).

**Figure 5:**
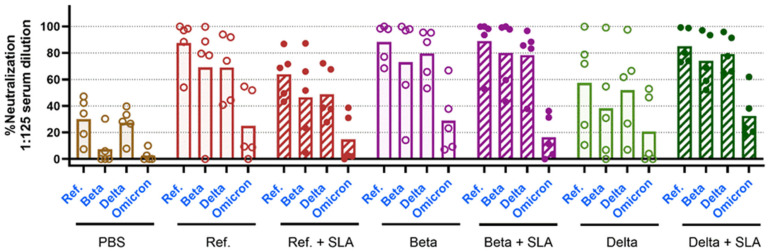
All booster formulations induce similar neutralization against Ref., Beta, and Delta but markedly less against Omicron 8 weeks after boosting. Female Balb/c mice, aged 8–10 weeks, were immunized with Ref. spike protein and SLA archaeosomes on days 0 and 21. On day 91, mice were immunized with the vaccine formulation, as indicated in black on the bottom of the graph. Blood samples were collected on day 147, and neutralization activity was assessed at 1:125 dilution against Ref., Beta, Delta, or Omicron spike protein, as indicated in blue on the graph. Bars are presented as means with individual data points scattered.

**Figure A2:**
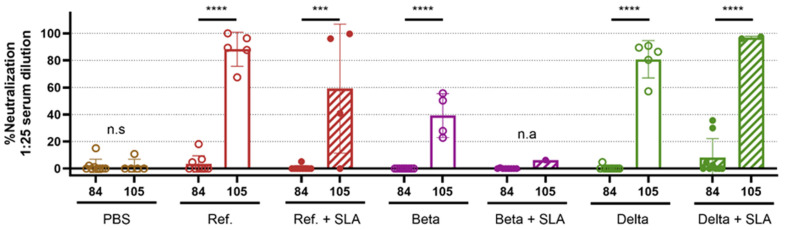
Boosting aged mice with a Beta-based vaccine results in reduced neutralization against the Ref. spike protein compared to other vaccines. Female Balb/c mice aged 19 months were immunized with the Ref. spike protein and SLA on days 0 and 21. Mice were boosted on day 91 with PBS or vaccine formulation, as indicated on the graph. Blood samples were collected at days 43, 84, and 105 and assessed for % neutralization against Ref. spike protein using a surrogate cell-based neutralization assay. Bars: Data is presented as mean ± SD and multiple unpaired *t* tests (two-stage step up, Benjamini, Krieger, and Yekutieli); not applicable (n.a.): too few mice remained; n.s. > 0.05, *** *p* < 0.005, and **** *p* < 0.001.

Sera collected on day 147, eight weeks after booster immunization, from all vaccine groups still demonstrated neutralization (Figure 5). All vaccines had a similar ranking of neutralization activity, with the highest neutralization against Ref. spike, slightly lower neutralization against Beta and Delta, and much weaker responses seen against Omicron, indicating that the variant antigen boosters did not improve responses to the corresponding spike and failed to overcome immune imprinting. For example, the Ref. booster vaccine effectively induces antibodies that cross-neutralize Beta (69%) and Delta (69%) spike protein–ACE2 binding at levels slightly lower (but not statistically significant) than Ref. spike (87%) (*p* > 0.05). However, neutralization against the Omicron variant was significantly lower (25%, *p* < 0.0001) (Figure 5). Interestingly, among aged mice, the Beta variant booster induced significantly less neutralization across the tested spike proteins compared to the Ref.- or Delta-based formulations (Figure A2).

**Figure 6:**
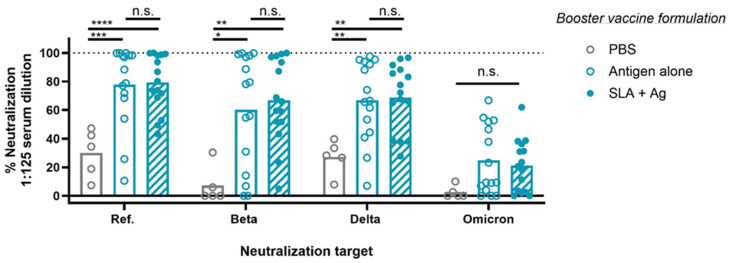
The inclusion of the adjuvant does not improve neutralization against Ref., Beta, Delta, or Omicron spike compared to antigen-alone formulations. Female Balb/c mice, aged 8–10 weeks, were immunized with Ref. spike protein and SLA archaeosomes on days 0 and 21. On day 91, mice were immunized with spike protein (Ref., Beta, or Delta) alone or with SLA archaeosomes, as indicated in the legend, or PBS control. Blood samples were collected on day 147, and neutralization activity was assessed against Ref., Beta, Delta, or Omicron spike protein, as indicated in blue on the graph. Bars are presented as means with individual data points scattered. One-way ANOVA with Tukey’s multiple-comparison test; n.s. *p* > 0.05, * *p* < 0.05, ** *p* < 0.01, *** *p* < 0.001, and **** *p* < 0.0001.

To evaluate whether the inclusion of SLA archaeosomes in the third vaccine dose enhances antibody neutralization, we compared the neutralization values from groups receiving spike protein alone (Ref., Beta, or Delta) to those from groups receiving the same spike protein combined with SLA archaeosomes (Figure 6). Both spike protein alone and SLA archaeosome-adjuvanted vaccines similarly improved neutralization compared to PBS control. Specifically, the neutralization against Ref. spike protein–ACE2 binding was enhanced from 30% in the PBS group to 78% and 79% for spike protein alone and SLA-adjuvanted groups, respectively (*p* < 0.001). For Beta and Delta spikes, neutralization activities of 7 and 20% were measured in the PBS group vs. 60 and 67% and 67 and 69% following immunization with spike protein alone and SLA-adjuvanted groups, respectively. Omicron spike neutralization increased from 3% to 25% with spike protein alone and 21% with SLA-adjuvanted groups. Overall, inclusion of SLA archaeosomes in the vaccine booster did not significantly enhance neutralization against spike protein compared to the unadjuvanted booster. Notably, the neutralizing activity against the Omicron spike protein was lower compared to the activity observed against the more closely related Ref., Beta, and Delta spike protein.

### 3.4. SLA Archaeosomes Enhance Cellular Responses Against SARS-CoV2 Spike Protein

**Figure 7:**
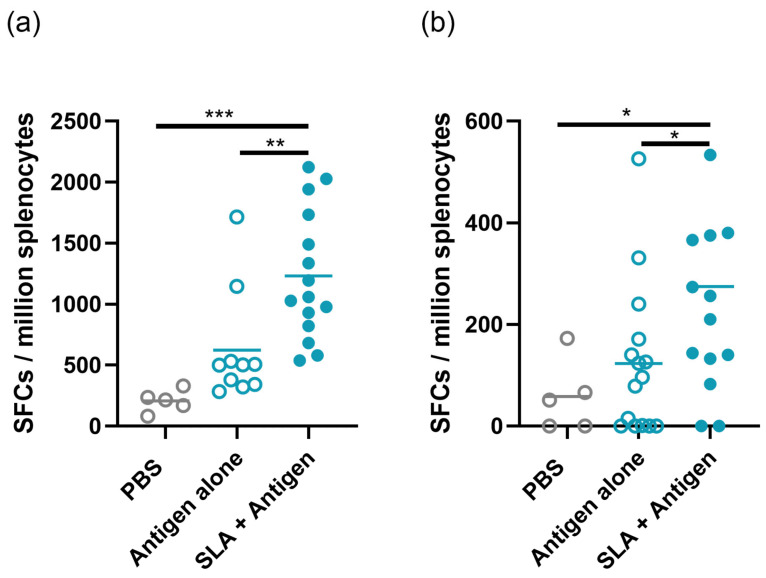
SLA-adjuvanted booster vaccines induce more IFN-γ-producing T cells as evaluated by ELISpot. Female Balb/c mice, aged (**a**) 8–10 weeks or (**b**) 19 months, were immunized with Ref. spike protein alone or with SLA archaeosome on days 0 and 21. On day 91, mice were immunized with spike protein (Ref., Beta, or Delta) alone (*n* = 10) or with SLA archaeosomes (*n* = 15), as indicated in the graph, or PBS control (*n* = 5). Data are presented as means with individual data points scattered, and vaccine groups were combined for comparison between antigen-alone or adjuvanted formulations. Splenocytes were collected 7 days post-booster immunization, stimulated with spike Ref.-derived peptides, and assessed by ELISpot for IFN-γ SFCs. One-way ANOVA with Tukey’s multiple-comparison test; * *p* < 0.05, ** *p* < 0.01, and *** *p* < 0.001.

**Figure A3:**
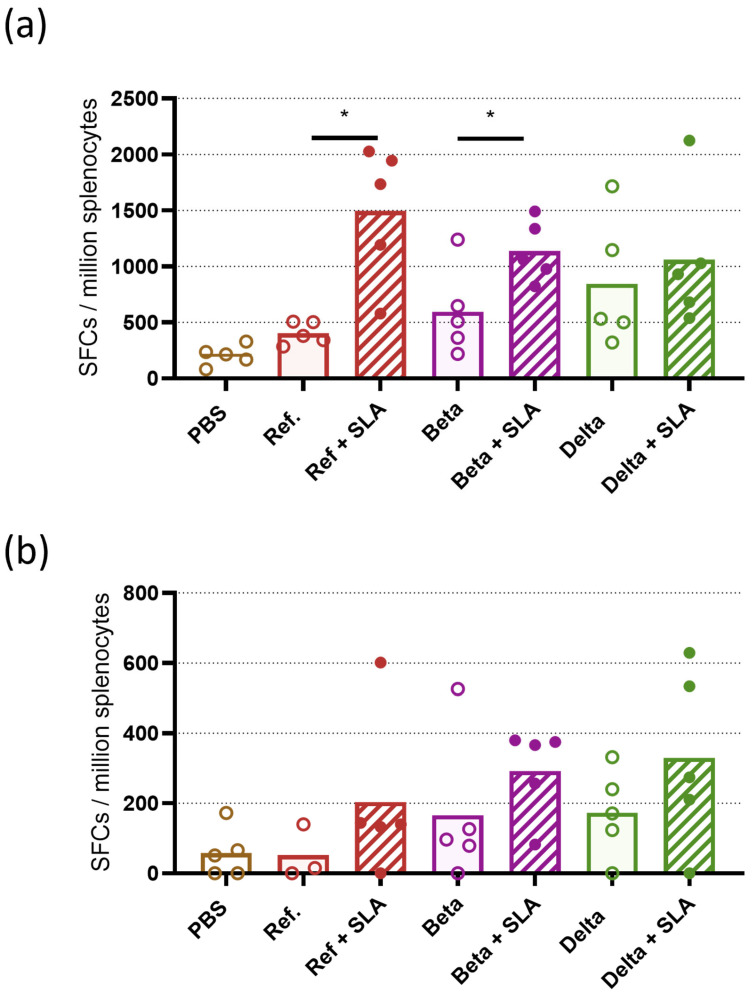
Addition of SLA to vaccine formulation enhances IFN-γ-producing T cells as evaluated by ELISpot for Ref. and Beta vaccinated young mice. Female Balb/c mice, aged (**a**) 8–10 weeks or (**b**) 19 months, were immunized with SLA archaeosome on days 0 and 21. On day 91, mice were immunized with spike protein (Ref., Beta, or Delta) alone (*n* = 10) or with SLA archaeosomes (*n* = 15), as indicated in the graph, or PBS control (*n* = 5). Vaccine groups were combined for analysis to compare antigen-alone or adjuvanted formulations. Splenocytes were collected 7 days post-booster immunization, stimulated with spike Ref.-derived peptides, and assessed by ELISpot for IFN-γ production spot-forming units (SFCs). For young mice immunized with a Ref.- or Beta-containing vaccine, the addition of SLA induced more IFN-γ producing T cells compared to antigen alone (Ref. *p* < 0.05; Beta. *p* < 0.05) or PBS (Ref. *p* < 0.01; Beta *p* < 0.05); the same was not observed among Delta-vaccinated mice nor any of the aged mice. Data is presented as mean with individual data points scattered, and a one-way ANOVA with Tukey’s multiple-comparison test was used. * *p* < 0.05.

To evaluate the ability of various vaccine regimens to induce spike-specific T cells, splenocytes were collected on day 98, seven days after the booster vaccination, and evaluated by IFN-γ ELISpot. The inclusion of SLA archaeosomes elicited a robust cellular response in young and aged mice, enhancing the number of spike-specific IFN-γ^+^ SFCs among Ref.- and Beta-boosted mice compared to antigen alone or PBS (Figure A3). For analysis, the values obtained with the various antigens were combined into two groups, i.e., with or without SLA archaeosome adjuvant (Figure 7). We observed that SLA archaeosome-adjuvanted vaccine formulations induced a significantly greater number of IFN-γ-producing cells compared to their antigen-alone counterparts and PBS control in both young (Figure 7a) and aged immunized mice (Figure 7b). Specifically, young mice that received an SLA archaeosome-adjuvanted booster vaccine induced, on average, 1231 SFCs, which was significantly greater than the antigen-alone booster’s 623 SFCs (*p* < 0.01) and the PBS control’s 206 SFCs (*p* < 0.005). In aged mice, a similar trend was observed, but at a substantially reduced overall number of SFCs. The SLA archaeosome-adjuvanted booster vaccine induced, on average, 275 SFCs, which was significantly greater than the antigen-alone booster’s 123 SFCs (*p* < 0.05) and the PBS control’s 58 SFCs (*p* < 0.05). These results underscore the enhanced immunogenicity of SLA archaeosome-adjuvanted vaccine formulations in inducing spike-specific cellular responses, particularly in terms of IFN-γ production, across both young and aged groups.

## 4. Discussion

The emergence and global spread of the SARS-CoV-2 virus have led to an unprecedented effort in vaccine development. Multiple vaccines have been licensed for emergency use to combat the spread of the virus. However, the emergence of highly transmissible viral variants that evade antibody responses has necessitated the continued testing of current vaccines and further development of new variant vaccines or vaccine strategies.

Herein, we set out to determine the ability of various booster regimens to enhance humoral and cellular responses to SARS-CoV-2 spike, whether variant-based antigens could overcome imprinting, and lastly, whether the inclusion of SLA archaeosomes could enhance responses. Following a priming series spaced 3 weeks apart using SLA archaeosome-adjuvanted Ref. spike protein, we induced a high level of spike-specific neutralizing antibodies in Balb/c mice. The same mice were boosted ten weeks later with either the same Ref. spike protein antigen or antigens based on variants of concern and Beta or Delta spike protein alone or in combination with the SLA archaeosome adjuvant. A single booster dose was administered to mimic the current vaccination strategy utilized clinically.

Antibody titers, neutralization, and IFN-γ-producing cellular responses were then assessed at various timepoints. Booster vaccines using Ref., Beta, or Delta spike protein all induced similar increases in antibody titers. These titers waned at a similar rate over the 17-month observation period, and all were capable of strongly neutralizing the binding of Ref., Beta, or Delta spike protein to ACE2. Although the neutralization of receptor binding to an Omicron spike protein was detectable, it was lower compared to the other variants. Overall, the use of a variant-based booster did not appear to improve the neutralizing activity against that particular variant’s spike when compared to a booster with Ref. spike protein.

In addition to testing the impact of antigen sequences on the booster vaccine, we evaluated the impact of including an adjuvant in the booster dose. Overall, there was no clear benefit of including the SLA archaeosome adjuvant on antibody levels or neutralization in the short term; however, it did result in enhanced antigen-specific cellular responses compared to boosting with an antigen alone. Given the known role of CD4 T cells in supporting long-term antibody maintenance, the observed increase in T cell responses may contribute to greater antibody durability. Indeed, our data show that adding SLA to the booster generated longer-lasting antibody responses compared to PBS control. Boosting with antigen alone generated similar kinetics of antibody response; however, the response waned faster than the SLA-adjuvanted sample (except for the antigen-alone Ref. sample, which was similar to the SLA-adjuvanted groups). This trend suggests that a protein booster alone could be particularly useful for boosting an existing antibody response, but when variant-based antigens are used, the inclusion of SLA archaeosomes could be beneficial for boosting longer-lasting antibody responses.

Early pre-clinical studies in non-human primates identified the correlate of vaccine protection for SARS-CoV-2 challenge to be high levels of antibodies capable of neutralization and/or other functions [29,45,46]. The adoptive transfer of a high dose of purified IgG can also block infection [47,48,49], which has prompted vaccine developers to advance candidates that induce a high titer of robustly neutralizing antibody with an overall goal of preventing infection and blocking transmission. An SLA archaeosome-adjuvanted vaccine induces a high level of neutralizing antibodies, and even a single dose has been shown to be highly efficacious in a SARS-CoV-2 hamster challenge model [33].

A major concern has been the limited durability of protection induced by current vaccines. For example, clinical trial and real-world data with BNT162b2 (Pfizer), mRNA-1273 (Moderna), and Ad26.COV2.S (AstraZeneca) has shown that, while high initial antibody titers were generated, titers waned over time and required booster immunizations within 8 to 12 months to maintain a high level of protective antibodies [50,51,52]. With the spread of each SARS-CoV-2 variant, protection was found to wane even more quickly such that after a fourth dose of a Ref.-spike-based vaccine (BNT162b2), protection from infection against the SARS-CoV-2 Omicron variant waned after only 4 weeks [53]. To address this, variant-specific vaccines have been developed. However, even after an Omicron-specific mRNA vaccine was administered as a booster in non-human primates, it was found to be no more effective than boosting with the original mRNA-1273 vaccine for protection against Omicron challenge [54]. Moreover, another study with a SARS-CoV-2-based vaccine has shown that a single booster dose may not be sufficient to overcome immunological imprinting and that a dual-vaccination approach with a variant-based vaccine was required to induce higher neutralization activity towards the corresponding variant [27].

In order to offset the limited duration of protection induced by current vaccines, more frequent booster vaccinations have been proposed for high-risk individuals. These boosters could help maintain high titers of antibody and prevent infection and transmission of the virus. However, this approach may potentially reduce overall vaccine uptake [55,56,57].

Our findings show that SLA archaeosomes in the booster dose enhanced cellular immune responses. Our previous research has shown that a spike–SLA vaccine induces spike-specific CD4 T cell responses [33]; this could explain the slightly prolonged antibody response observed with SLA at day 525, as CD4 T cells are known for their ability to support the long-term maintenance of antibody-producing B cells [58]. Since existing vaccines might not be able to prevent the infection or transmission of novel variants of concern, requiring more frequent booster doses, an alternative approach is to reconsider the qualities of an ideal SARS-CoV-2 vaccine. While the short-lived neutralizing antibody responses induced by current vaccines may be beneficial for reducing initial infection and preventing severe disease, a robust cellular response (involving B and T cells) is likely necessary for prolonged protection, preventing disease progression, and reducing hospitalization rates [55,59].

SLA archaeosome-adjuvanted vaccines have a proven track record to induce both a robust antibody and CD8 T cell response that can be expanded with repeat SLA archaeosome-adjuvanted boosters. For example, we have previously demonstrated that, when used as an adjuvant to hepatitis B antigen (HBsAg), SLA archaeosomes were able to induce a greater frequency of CD8 T cells as well as greater in vivo cytotoxicity compared to various other adjuvants that include TLR3/4/9 agonists, oil-in-water and water-in-oil emulsions, and aluminum hydroxide [36].

The role of CD8 T cells in SARS-CoV-2 immunity is arguably only partially understood. Indeed, neutralizing antibody responses were deemed to be essential for SARS-CoV-2 protection, and in early SARS-CoV-2 infection and CD8 T cell depletion studies, it was shown that when sufficient antibody is present, the absence of CD8 T cells only partially impaired protection [49,60], and the passive transfer of convalescent antibodies could provide both prophylactic and therapeutic protection [47,48]. However, it was later observed that during the Omicron surge in South Africa, both BNT162b2 and Ad26.COV.2 (Ref.-based vaccines) imparted robust protection from hospitalization and death despite the absence of high titers of Omicron-neutralizing antibodies, suggesting a role for other immune responses, such as non-neutralizing antibodies and T cells, in providing cross-variant protection from Omicron [55].

While it is clear that neutralizing antibody responses are a critical determinant of protection, T cells also play an essential role in viral clearance, providing memory and also recognizing and protecting against viral variants that evade antibody neutralization [61]. The additional layer of protection provided by T cells may also prove indispensable in protecting the elderly, who often have significantly diminished levels of neutralizing antibody. Given that SLA archaeosome-adjuvanted vaccines induce strong CD8 T cell responses, this could be a critical advantage in scenarios where neutralizing antibody titers are insufficient, such as in elderly populations or during outbreaks of immune-evasive variants.

While our findings highlight important qualities of SLA archaeosome-adjuvanted vaccines to induce and boost immunity towards SARS-CoV-2, there are some limitations that should be considered. This study was conducted solely with female Balb/c mice; as sex hormones can influence both humoral and cellular immunity, it is an important consideration to note. While SLA archaeosomes enhanced cellular responses, they did not significantly improve cross-variant neutralizing antibody activity. This could be due to immunological imprinting that limited de novo B cell activation toward variant-specific epitopes, or it could relate to the antigen presentation pathways stimulated by SLA, a comparison with other adjuvants would be interesting. While our previous studies have shown that the assessment of antibody neutralization using Vero E6 cells correlates well with the results obtained from plaque reduction neutralization tests (PRNTs), pseudolentiviral NT50, and even viral challenge within a hamster model [33,34], it is important to note that alternative cells are commonly used in pseudo-neutralization assays (HEK-293T-ACE2 or ACE2 + TMPRSS2-expressing cells). Future studies will include viral challenge experiments and assessment of neutralizing antibody responses in respiratory compartments such as bronchoalveolar lavage fluid and nasal washes. Evaluating mucosal immunity is particularly important for respiratory pathogens like SARS-CoV-2, as it reflects a vaccine’s potential to limit viral replication and droplet-based transmission. Further work will also examine the synergy of SLA archaeosomes with Toll-like receptor (TLR) agonists such as Poly(I:C) and CpG, which have previously been shown to enhance both humoral and cellular responses [62] and could be used as a strategy to broaden protection towards variants of concern or perhaps overcome immune imprinting with a single booster dose.

## 5. Conclusions

In conclusion, both protein alone and SLA archaeosome-adjuvanted spike antigen boosters have important but distinct roles in SARS-CoV-2 vaccination strategies. Protein subunit boosters offer a simplified and accessible formulation and were effective at amplifying existing humoral responses, making them well-suited for boosting immunity in individuals already primed against the reference strain. In contrast, SLA archaeosome-adjuvanted formulations were superior in enhancing spike-specific T cell responses, which are likely to play a critical role in initiating de novo immunity to new variants of concern and in supporting the long-term maintenance of antibody responses. While SLA archaeosomes did not overcome immune imprinting in our study, they did induce cross-reactive antibodies to multiple SARS-CoV-2 variant spikes, and their ability to enhance cellular immunity may be especially valuable in scenarios where sustained protection is needed. Our findings also align with previous reports suggesting that a single-dose boost regimen may be insufficient to redirect immunity toward variant antigens, indicating that strategies such as dual boosting could be explored. Future work should continue to evaluate adjuvants like SLA archaeosomes that can be paired with diverse antigens to generate robust, long-lived cellular responses and potentially extend antibody durability, thereby strengthening preparedness against emerging variants and future pandemics.

## 6. Patents

FCS, BA, GA, UDH, SR, and MJM are inventors on various SLA-related patents. YD is an inventor of a patent application related to human resistin-fused spike antigens. All other authors declare no conflicts of interest, nor competing financial interests.

## Figures and Tables

**Table 1:** Immunization groups.

Groups	1st and 2nd Immunization on Days 0 and 21	3rd Immunization on Day 91
1	Ref. spike protein + SLA (*n* = 70)	PBS
2		Ref. spike protein (*n* = 10)
3		Beta spike protein (*n* = 10)
4		Delta spike protein (*n* = 10)
5		Ref. spike protein + SLA (*n* = 10)
6		Beta spike protein + SLA (*n* = 10)
7		Delta spike protein + SLA (*n* = 10)

## Data Availability

The raw data supporting the conclusions of this article will be made available by the authors on request.

## Abbreviations

The following abbreviations are used in this manuscript:

SLA	Sulfated lactosyl archaeol (a glycolipid-based archaeosome adjuvant);
Ref.	Reference spike protein (ancestral strain SARS-CoV-2 spike);
CHO	Chinese hamster ovary (cell line used for protein expression);
SFCs	Spot-forming cells (in ELISpot assay);
HA	Hemagglutinin (influenza antigen);
HBsAg	Hepatitis B surface antigen;
HCV	Hepatitis C virus;
RHDV	Rabbit hemorrhagic disease virus;
SmCB	Schistosoma mansoni cathepsin B antigen.
